# Interferon stimulatory DNA activates the DNA damage signaling through ATM and DNA-PK sensing

**DOI:** 10.1016/j.jbc.2026.111362

**Published:** 2026-03-09

**Authors:** Samira Kemiha, Lorena Rejón-Franco, Estelle Ghibaudo, Roger J. Eloiflin, Morgane Chemarin, Karim Hawillo, Nadine Laguette, Hervé Técher

**Affiliations:** 1 Université Côte d’Azur, IRCAN, CNRS, INSERM, Nice, France; 2 IGMM, Université de Montpellier, CNRS, Montpellier, France

**Keywords:** ATM, cGAS-STING pathway, DNA damage response, DNA-PK, Interferon stimulatory DNA

## Abstract

In eukaryotic cells, DNA is normally confined in the nucleus and mitochondria and the presence of DNA in the cytoplasm is a danger signal that activates innate immune responses. Upon detection of cytoplasmic dsDNA in mammalian cells, the cGAS-STING pathway induces type I-Interferon and inflammatory responses, a key step in innate immune activation. Since its discovery, Interferon Stimulatory DNA (ISD), a linear double-stranded DNA, has been largely used to study the cGAS-STING pathway and its regulation. Here, we show that ISD also stimulates DNA damage signaling. We show that ISD activates both ataxia telangiectasia mutated and DNA-dependent protein kinase, the sensor kinases of the DNA damage response, independently of cGAS-STING signaling. Our results demonstrate that the DNA damage response, which is usually considered a response to genomic DNA lesions, can be promoted by foreign DNA. Our data further suggest that ISDs coordinate two central protective functions of cells, the innate immunity and DNA damage checkpoints.

The cyclic GMP-AMP synthase stimulator of interferon gene (cGAS-STING) pathway is a part of innate immunity and contributes to protect cells and organisms against pathogenic infections and damage. This protection is mediated by inflammation, which is an alert response to the detection of damage- or pathogen-associated molecular patterns, including immune-stimulatory DNA of endogenous or exogenous origin. The protein cGAS is a sensor of double-stranded (ds) DNA (1). Upon recognition of dsDNA, cGAS synthesizes the 2′3′-cGAMP cyclic dinucleotide. 2′3′-cGAMP binds to the STING adaptor protein that subsequently assembles a signalosome comprised of Tank-binding kinase 1 (TBK1) and Interferon Regulatory Factor 3. Phosphorylation of STING and Interferon Regulatory Factor 3 promotes type-I Interferon responses (IFN-I, characterized by the production of IFN-α and IFN-β). Recognition of DNA by the cGAS-STING pathway is currently accepted to mostly rely on its subcellular compartmentalization, that is in the cytoplasm *versus* in the nucleus or mitochondria in normal conditions (2). In addition to non-self cytosolic dsDNA species, self-DNA from mitochondria or unstable genomes have all been shown to activate cGAS and to contribute to a large panel of auto-immune and inflammatory pathologies, as well as to cellular senescence and aging (2, 3). The cGAS-STING pathway has also been proposed to be a target of choice in cancer therapies, where boosting STING-dependent inflammation may promote antitumoral responses (4, 5). It is therefore essential to understand how cells respond to cytosolic DNA and the regulation of cGAS-STING signaling.

An important tool available to investigate cGAS-STING signaling is the transfection of the so-called Interferon Stimulatory DNA (ISD) (6). This dsDNA oligo of 45 base pairs was derived from *Listeria monocytogenes* genome. It does not contain CpG sequences and has the particularity of promoting a potent IFN-I response, in a cGAS- and STING-dependent manner, when transfected into mammalian cells (6, 7). In addition to ISD, cGAS activation has been achieved with different sequences of dsDNA, notably poly (dA:dT), herring testes DNA or calf thymus DNA (6, 7, 8, 9).

There is a tight connection between genomic instability and cGAS-STING signaling. For instance, the cGAS-STING signaling can be activated by various DNA damaging conditions, which expose dsDNA in the cytosol of challenged-cells (10, 11, 12, 13, 14, 15). More recently, we have proposed that IFN-I and STING-signaling may be sufficient to generate DNA damage and to alter the proliferation of human fibroblasts and epithelial cells (16). Previous findings, including ours, have reported that inactivation of the cytoplasmic exonuclease TREX1, known to be deficient in several auto-inflammatory disorders, leads to DNA damage, chromosomal instability and senescence (16, 17, 18). These results were recently supported by the observation that STING auto-activating mutations, which are known to cause the STING-associated vasculopathy with onset in infancy, lead to DNA damage accumulation (19). Altogether, these data showed that STING-signaling and the IFN-I response can generate DNA damage and chromosomal instability and is tightly intertwined with DNA damage response. Furthermore, a major sensor of DNA breaks, the DNA-dependent protein kinase (DNA-PK) of the DNA damage response (DDR) has been shown to detect cytosolic dsDNAs and to act as a regulator of the IFN-I pathway (9, 20, 21, 22, 23). We therefore investigated whether ISD may activate DNA damage signaling.

## Results

### ISD transfection leads to phosphorylation of histone H2AX

**Figure 1:**
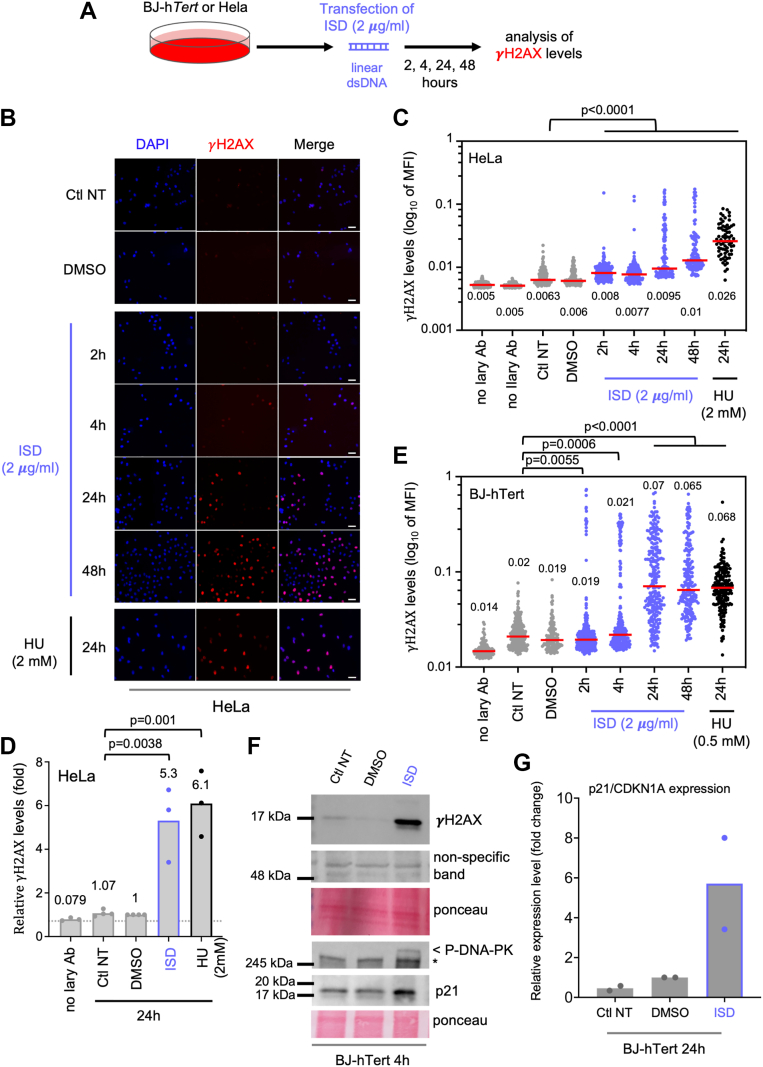
**Transfection of ISD leads to DNA damage signaling.***A*, HeLa and BJ-hTERT cells were transfected with 2 μg/ml of ISD (linear dsDNA) for 2, 4, 24 or 48 hours (h), then γH2AX was monitored by immunofluorescence. *B*, representative images of γH2AX staining (*red*) in HeLa cells transfected or not with 2 μg/ml ISD for 2, 4, 24 and 48 h. Hydroxyurea was used as a positive control. Nuclei were stained with DAPI (*blue*). The scale bar represents 100 μm. *C*, quantification of nuclear levels of γH2AX intensity (mean fluorescence intensity) is shown from a representative experiment in HeLa cells. Background levels were assessed performing the staining without primary (no Iary) or secondary antibodies (no IIary). Each dot shows the mean fluorescence intensity in an individual nucleus. The horizontal red line shows the median for each condition and is indicated. Around 100 nuclei or more were quantified in each condition. Non-parametric Mann and Whitney test was used to compare distributions and *p*-values are shown. *D*, HeLa cells were transfected for 24 h with 2 μg/ml of ISD and levels of γH2AX were quantified by fluorescent microscopy, as in *C*. Bars are the relative mean intensity (fold change) obtained from independent experiments. The relative fold change is indicated. Each dot is the mean relative intensity obtained from an independent experiment. *p*-values are the results of unpaired *t* test. *E*, quantification of nuclear levels of γH2AX is shown from a representative experiment in BJ-h*TERT* cells. Results are presented as in C. *F*, Western blot analysis of γH2AX in BJ-h*TERT* cells transfected or not with 2 μg/ml ISD. Non-specific band and Ponceau are shown as loading controls. Similar results were obtained in HeLa cells (shown in Fig. S1*C*). *G*, BJ-hTERT cells were transfected or not with 2 μg/ml ISD for 24 h. p21/CDKN1A mRNA levels were quantified by RT–qPCR. Results are the mean of two independent experiments. The experiments shown are all representative of at least two independent experiments. γH2AX, phosphorylation of the histone H2AX; ISD, interferon stimulatory DNA

To assess DDR activation upon ISD transfection, we first assessed phosphorylation of the histone H2AX (γH2AX). γH2AX is a classical DNA damage marker induced by a variety of DNA damages, notably upon recognition of DNA breaks in an Ataxia telangiectasia mutated (ATM) or DNA-PK dependent manner (24). We transfected human immortalized cervical carcinoma-derived cancer cells (HeLa) and immortalized BJ-h*TERT* fibroblasts with ISD and assessed γH2AX levels by immunofluorescence (Fig. 1*A*). In most experiments, treatment with the replication stress inducer hydroxyurea (HU) was used as a positive control, as previously characterized (25, 26), while negative controls were non-treated and non-transfected cells (thereafter Ctl NT) and cells exposed to DMSO (Fig. 1, *B* and *C*). In HeLa cells, we observed a drastic increase of nuclear γH2AX signal at 24 and 48 h post-transfection of ISD, as compared to non-transfected cells (Fig. 1, *B* and *C*). We quantified nuclear γH2AX intensity and consistently observed a significant increase of γH2AX intensity 24 and 48 h after transfection of ISD in HeLa cells (Fig. 1*D*). The five-fold increase of γH2AX levels induced by ISD at 24 h was comparable to that measured in HU-treated cells (Fig. 1*D*). Similar data were obtained in BJ-h*TERT* cells, where we observed a potent γH2AX signal 24 and 48 h post-transfection as compared to control non-transfected cells (Fig. 1*E*). Similarly to what was observed in HeLa cells, a five-fold γH2AX induction at 24 h post-ISD stimulation was observed, and it was comparable to that induced in response to HU treatment (Figs. 1*E* and S1*A*).

We next assessed the concentration of ISD required to activate the DDR signaling. We transfected BJ-h*TERT* cells with a range of ISD concentrations from 0.01 μg/ml to 2 μg/ml for 24 h, compared to MOCK transfection. We observed that γH2AX signal is strongly detected when cells are transfected with quantities of ISD ranging from 0.5 to 2 μg/ml (Fig. S1*B*). Low amount of 0.25 μg/ml of ISD induced a limited phosphorylation of histone H2AX, while the lowest tested concentrations (0.1 and 0.01 μg/ml) did not activate a detectable DDR-signaling (Fig. S1*B*). This suggests that the threshold of activation of DNA damage response following ISD transfection is above 0.25 μg/ml.

A tendency to induce γH2AX signal was observed as early as 4 h following ISD transfection in both HeLa and BJ-h*TERT* cells (Fig. 1, *C* and *E*). These results suggest that the DNA damage signaling is triggered shortly after ISD transfection. Using Western blot analyses, we confirmed that 4 h of ISD transfection was sufficient to induce a detectable increase of γH2AX levels in both HeLa (Fig. S1*C*) and BJ-h*TERT* cells (Fig. 1*F*). Together, these data indicate that ISD transfection can lead to DNA damage response activation as early as 4 h post-transfection, which then may increase in intensity, and persists for at least 48 h. To confirm that ISD induces DDR signaling, we assessed phosphorylation of apical kinases of the DDR, ATM and DNA-PK, as well as the protein level of downstream cell-cycle inhibitor p21. The *p21* gene is induced by DDR to inhibit cell-cycle progression in response to DNA damage (27). We observed that 4 h post-transfection p21 accumulated and DNA-PK is activated in BJ-h*TERT* (Fig. 1*F*). Consistently, we also observed a drastic increase in expression of *p21* (encoded by CDKN1A) upon ISD transfection (Fig. 1*G*). We next assessed whether the ATM apical kinase that detects DNA double-strand breaks (28) was activated in this context. In THP-1 leukemia cells, ISD transfection for 24 h led to increase γH2AX and P-ATM signals (Fig. S1*D*). Kinetics experiments in BJ-h*TERT* showed that γH2AX peaks at 4 and 24 h post-transfection and then persist until 48 h (Fig. S1*E*). These results demonstrate DDR activation following ISD transfection (Figs. 1 and S1).

### ISD transfection leads to ATM and DNA-PK activation independently of the cGAS-STING pathway

**Figure 2:**
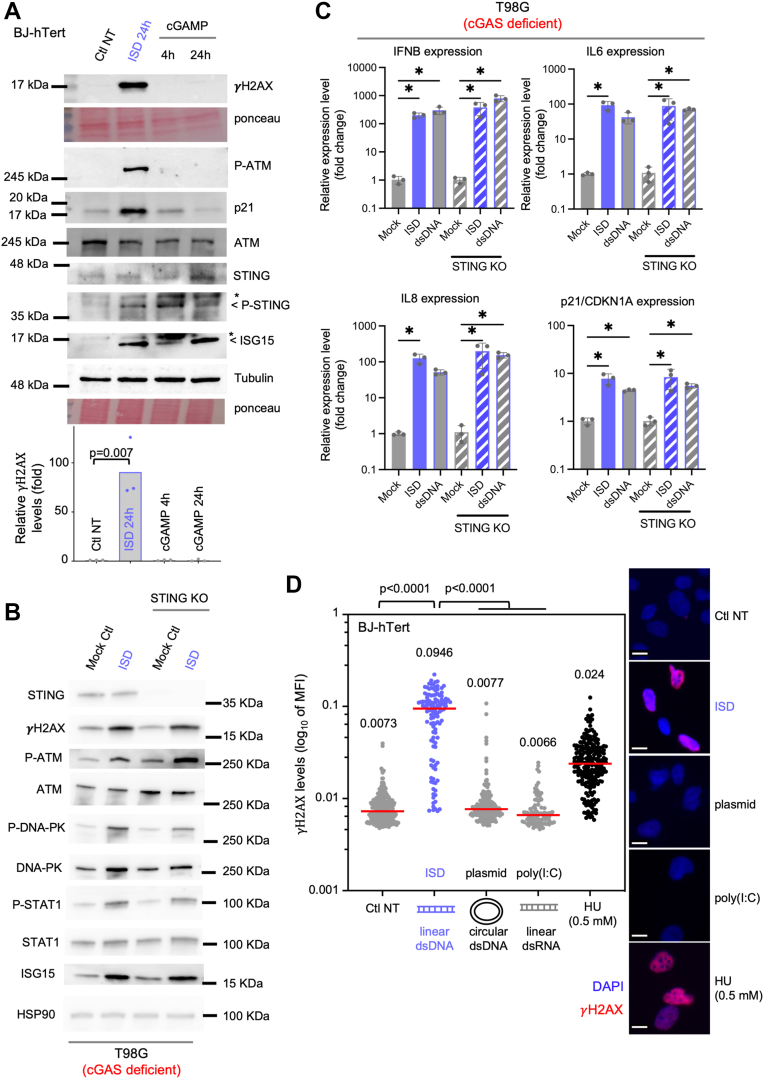
**ISD activates the ATM-DNA-PK pathway independently of the cGAS-STING pathway.***A*, Western blot analysis of DNA damage signaling in BJ-h*TERT* cells treated or not with 2 μg/ml ISD (24 h) and 20 μM of cGAMP (4 and 24 h). The experiment is representative of at least two independent experiments (see also Fig. S2*B*). Phosphorylation of STING and induction of ISG15 are internal controls of activation of STING signaling and IFN-response, respectively. Tubulin and Ponceau are used as loading controls. *Lower panel* shows the quantification of γH2AX signals from three independent experiments. *p*-value is the result of unpaired *t* test. *B*, Western blot analysis of DNA damage signaling in parental (cGAS deficient) and STING KO T98G cells transfected with 2 μg/ml ISD or not (MOCK Ctl). Results are presented as in *A*, at the difference that HSP90 is used as a loading control, and P-STAT1 is a marker of IFN-signaling. *C*, parental (cGAS deficient) and STING KO T98G cells were transfected or not with 2 μg/ml ISD or dsDNA for 24 h, as in *B*. Interferon-β, IL6, IL8 and p21/CDKN1A mRNA levels were quantified by RT–qPCR. Histograms are the mean of technical triplicates. Asterisks show the statistical significance obtained with one-way ANOVA. *D*, quantification of nuclear levels of γH2AX is shown from a representative experiment in BJ-hTERT cells. Results are presented as in Figure 1*C* and are representative of at least two independent experiments. ATM, ataxia telangiectasia mutated; γH2AX, phosphorylation of the histone H2AX; ISD, interferon stimulatory DNA

We next verified whether our ISD transfection was able to cause the expected activation of cGAS-STING-dependent prototypical type I IFN and inflammatory responses. To this aim, we assessed the expression of Interferon-β, of pro-inflammatory cytokine interleukin 6 (IL6) and of Interferon Stimulated Gene 15 (ISG15). ISD transfection led to increased expression of Interferon-β, *IL6* and *ISG15* in BJ-h*TERT* fibroblasts (Fig. S2*A*). We next assessed whether the transcriptional activation observed was reflected at the protein level. To this aim, we transfected BJ-h*TERT* cells for 24 h with ISD prior to Western blot analyses. We observed that ISD transfection led to ISG15 induction and STING phosphorylation (Figs. 2*A* and S1*E*).

Then we questioned whether the DNA damage response observed upon ISD transfection is dependent on the production of 2′3′-cGAMP upon detection by cGAS. To this aim, 2′3′-cGAMP was supplemented alongside ISD transfection to BJ-h*TERT* cells, prior to WB analyses. We found that although 2′3′-cGAMP treatment causes increased levels of hallmarks of STING activation comparable to ISD transfection, no γH2AX was detected (Fig. 2*A*). We found that ISD transfection, but not 2′3′-cGAMP stimulation, led to phosphorylation of ATM, phosphorylation of DNA-PK and accumulation of p21 (Figs. 2*A* and S2*B*). These data suggest that ISD activates the DDR signaling, notably ATM- and DNA-PK-axis, independently of 2′3′-cGAMP production by cGAS. This further suggests that STING-dependent IFN production is not sufficient to cause DNA damage response activation, in this context.

To formally assess the role of STING signaling in DDR activation in the presence of ISD, we next performed experiments where STING is inhibited in BJ-h*TERT* cells. We found that inhibition of STING did not suppress the level of γH2AX quantified by immunofluorescence (Fig. S3*A*). We then confirmed these results using T98G glioblastoma cell lines that are naturally deficient for cGAS, and in which STING knockout are available (T98G STING KO), as previously described (20). In parental T98G cells (cGAS deficient), IFN-I responses are induced by the DNA break sensor and kinase DNA-PK, independently of cGAS (20). We therefore assessed DNA damage signaling and IFN response induced by ISD in T98G and T98G STING KO cells. We observed that in both T98G and T98G STING KO, ISD led to IFN signaling, with increased levels of ISG15 and P-STAT1 (Fig. 2*B*), and transcription of IFN-β, IL8 and IL6 (Fig. 2*C*), consistent with previous findings (20). ISD transfection in T98G cells, either in STING proficient or STING KO, showed marked DNA damage signaling markers increase (Fig. 2*B*). We recapitulated the induction of γH2AX, P-DNA-PK and of P-ATM (see Figure 2*B* and associated quantifications in S3B) observed upon ISD transfection in BJ-h*TERT* and THP-1 cells. We further validated that STING ablation in THP-1 cells does not suppress γH2AX induction upon ISD transfection (Fig. S3*C*). Thus, altogether these results show that ISD activates both the ATM and DNA-PK apical kinases of the DDR independently of canonical STING signaling.

### Linear dsDNA induces DNA damage signaling

We next assessed whether the DNA damage signaling induced by ISD transfection is specific of ISD or can be induced by RNA or other DNA structures and sequences. We therefore transfected BJ-h*TERT* cells with ISD (linear dsDNA), with a plasmid (circular dsDNA), or with poly(I:C) (dsRNA). Poly(I:C) and circular DNA can activate inflammatory responses similar to that triggered by ISD, through RNA sensors or cGAS, respectively (6, 7, 9). We observed that only ISD, but not poly(I:C) nor plasmid DNA, induced γH2AX by immunofluorescence (Fig. 2*D*). Thus, neither circular DNA nor dsRNA are able to activate the DDR, suggesting that ISD could activate DDR through its exposed DNA ends, which mimic broken DNA. We observed that transfection with another sequence of linear dsDNA (hereafter called dsDNA, previously described in (8, 20, 29)) also activates ATM-signaling independently of cGAS and STING in T98G cells (Figs. S4 and S3*B*). We further validated these findings by analyzing by RT-qPCR the expression level of IFN-responsive genes and of p21-coding gene, CDKN1A, which is induced by the DDR. Our results show that IFN-I and inflammatory responses, as well as DDR, including p21 are all induced by both ISD and dsDNA in cGAS-deficient T98G cells, upon ablation of STING or not (Figs. 2, *B* and *C*, S3*B*, S4). In summary, our results confirm that the activation of cGAS- and STING-independent sensors drives the activation of type I IFN and inflammatory responses, notably in absence of cGAS and STING (Fig. 2, *B* and *C*, and (9, 20, 21)). These alternative sensors are likely apical kinases of the DDR pathway, ATM and DNA-PK, which may also participate to the observed DNA damage response signaling.

### DDR signaling in response to ISD transfection is ATM- and DNA-PK-dependent

**Figure 3:**
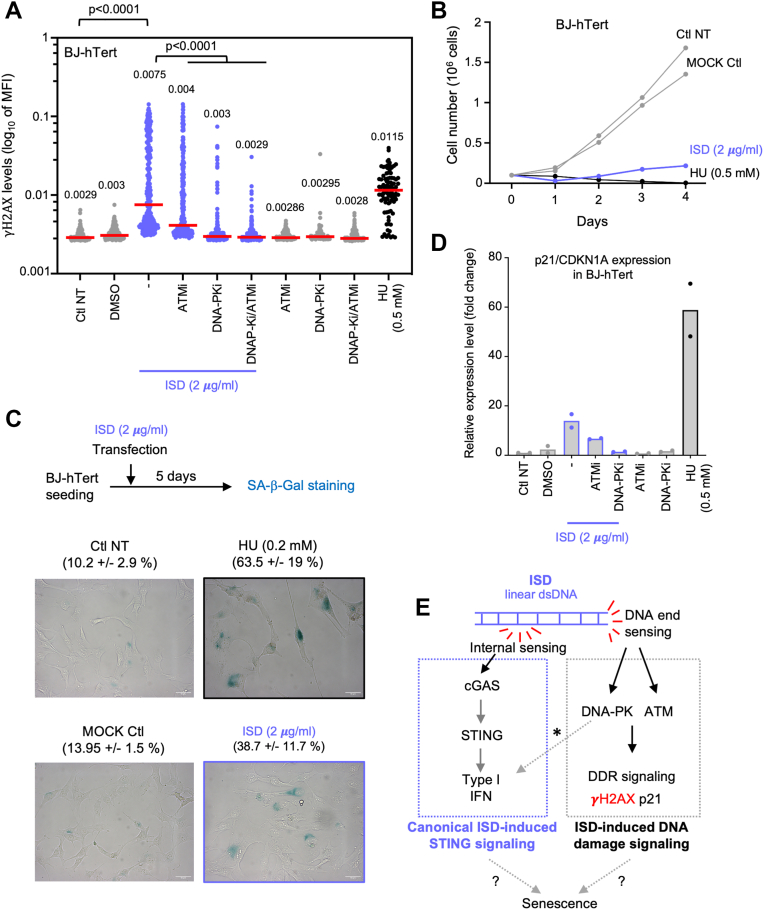
**DNA-damage signaling induced by ISD is mediated by both ATM and DNA-PK kinases.***A*, BJ-hTERT cells were treated with 2 μM of ATM or DNA-PK inhibitors (i) 1 h prior to transfection with 2 μg/ml ISD for 24 h. Quantification of nuclear levels of γH2AX are shown as in Figure 1*C*. Results are from one representative experiment (n = 2). *B*, the proliferation of BJ-h*TERT* fibroblasts was assessed for four consecutive days, following MOCK or ISD transfection. *C*, senescence-associated-β-galactosidase staining was performed 5 days after ISD transfection. Mean percentage and standard deviation for each condition is indicated (n = 2). *A*, *B* and *C*, hydroxyurea was used as positive control. One representative result is shown from at least two independent experiments. *D*, in BJ-hTERT cells, p21/CDKN1A mRNA levels were quantified by RT–qPCR in experimental conditions described in Figure 3*A* (n = 2). *E*, our work suggests the following model. ISD, or other types of linear dsDNA, are sensed internally by cGAS and at DNA ends by DNA-PK and ATM. Activation of cGAS by foreign DNA leads to STING signaling and IFN-I response. In parallel to this DNA sensing, DNA-PK and ATM detect ISD and activate the DNA damage response. We also know that DNA-PK can constitute an alternative DNA sensing pathway, which results in IFN-I response (shown by an *asterisk*, References 9 and 20). Our results suggest that the consequence of activation of both cGAS- and DDR-signaling is cell proliferation arrest and senescence. See further details in the discussion. ATM, ataxia telangiectasia mutated; γH2AX, phosphorylation of the histone H2AX; ISD, interferon stimulatory DNA.

Altogether, our results show that ISD, in addition to being sensed by cGAS to trigger IFN-I, is also sensed by DDR sensors to promote DNA damage signaling by ATM and DNA-PK kinases. We therefore assessed which kinase is responsible for DDR signaling upon ISD transfection. We pre-treated BJ-h*TERT* cells for 1 hour with ATM inhibitor (ATMi, KU55933 at 2 μM) and/or DNA-PKi (NU7441 at 2 μM). We next transfected ISD in presence of inhibitors and waited 24 h to analyze γH2AX levels by fluorescent microscopy. We found that both ATM and DNA-PK inhibitions impacted DDR-signaling. ATM inhibition partially decreased γH2AX levels, while DNA-PK inhibition suppressed it completely (Fig. 3*A*). These results show that the DNA-damage signaling induced by ISD is mediated by both ATM and DNA-PK kinases, with a prominent role for DNA-PK. Because cGAS-STING and DDR pathways are both required to promote senescence (16, 30, 31, 32, 33), we next assessed proliferation of BJ-h*TERT* cells after ISD transfection. We observed that, similarly to HU-treated cells, ISD-transfected cells do not grow (Fig. 3*B*). We then observed that ISD transfection led to an increase frequency of senescence-associated-β-galactosidase positive cells (Fig. 3*C*). Accordingly, we show here that foreign dsDNA leads to the expression of p21 (Figs. 1*F* and 2*C*), a well-known senescence marker (34). Using inhibitors, our results indicate that p21 induction in response to ISD is DNA-PK-dependent (Fig. 3*D*). Our results suggest that a consequence of activating both cGAS-STING and DDR pathways upon ISD transfection is senescence.

## Discussion

Our data show that the presence of foreign linear dsDNA is sufficient to activate DNA damage signaling. Our results support the conclusion that linear dsDNA, such as ISD, exposes DNA ends mediating a DNA-PK and ATM-dependent DNA damage signaling. ISD is a classical activator of the cGAS-STING pathway and therefore has been extensively used as an experimental condition to dissect cytosolic DNA-induced inflammation (6, 7, 9). Our work indicates that ISD can produce confounding effects by triggering the central signalization pathway of the DDR involved in cell-cycle regulation, DNA repair, apoptosis and senescence (27, 28). Earlier reports have shown the phosphorylation of ATM and histone H2AX upon transfection or micro-injection of cells with different dsDNA oligos (9, 20, 29, 35, 36). Accordingly, we report that ISD transfection results in proliferation arrest and senescence. Further work is required to decipher the molecular mechanism of this arrest of proliferation induced by foreign DNA.

Our data suggest three main routes by which ISD can lead to DDR signaling. First, exogenous linear dsDNA inside cells will mount an ATM- and DNA-PK-dependent DNA damage signaling. ISD or other linear dsDNA are structurally similar to DNA double-stranded breaks, exposing DNA ends that can be recognized by apical sensors of the DDR, namely DNA-PK and ATM (Fig. 3*E*). It has been previously reported in *Xenopus* egg extracts that linear DNA oligos, notably poly(dA:dT) sequences, are processed by the endo/exonuclease MRE11. This would lead to the generation of short ssDNA oligos that promote DNA damage signaling from the ATM axis of the pathway (36). Such a mechanism is likely involved in response to ISD transfection, although direct binding of DNA-PK to ISD extremities is presumably the main mode of activation of DDR in this case. In support of this later hypothesis, we observed that DNA-PK inhibition abrogates γH2AX signal, while ATM inactivation had only a partial effect.

The second hypothesis is that transfection of ISD produces a strong STING-signaling and IFN-I response, that in turn destabilizes the genomic DNA. We and others have previously shown that cGAS-STING signaling, and IFN-I contribute to generate replication stress and lead to the accumulation of DNA damage (16, 19, 37, 38). The fact that 4 h of ISD transfection are sufficient to detect the γH2AX signal and then later increase, suggested to us that this DNA damage response may depend on STING- and IFN-I-signaling, notably at later time points. However, up to 24 or 48 h of ISD transfection we observed histone H2AX and ATM phosphorylation in both T98G and HeLa cancer cell lines that are defective in cGAS and STING, respectively (20, 39, 40, 41). Therefore, the DNA damage signaling that we observed in T98G and HeLa cells is unlikely to be STING- and IFN-I -dependent. This conclusion is further confirmed by our results in STING KO T98G and THP-1 cells, showing that in absence of STING the DDR signaling is still activated. We therefore favor the hypothesis that DDR, in response to ISD, occurs in absence of genomic DNA lesions. In support of this later hypothesis, we do not observe the formation of 53BP1 foci, a sensitive DNA break marker, 2 to 48 h post-ISD transfection in HeLa cells (Fig. S5). We cannot exclude that a contribution of STING and IFN-I in generating a stress may occur after persistence of this response for longer time points. Indeed, chronic inflammation is pathologic. Recent findings suggest that uncontrolled STING activation leads to the accumulation of DNA damage and arrest of proliferation of cells by senescence, notably in inflammatory disorders, STING-associated vasculopathy with onset in infancy, AGS (Aicardi-Goutières Syndrome) and different forms of lupus (16, 17, 19, 38).

Thirdly, we cannot exclude that in addition to exposing DNA ends, ISD transfection leads to the titration of important chromatin and DNA repair factors. A large amount of DNA, once in the cytosol, may attract and interact with DNA binding factors. Some of these factors may have important roles in chromatin function and genome maintenance. For instance, it has been proposed that ssDNA can sequester key DNA repair and fork protection factors, namely RPA and RAD51 (18). Because RAD51 has been recently shown to have a dsDNA binding function (42, 43), we think that dsDNA oligos, such as ISD, can sequester RAD51. Similarly, the methyl CpG-binding protein 2 was recently shown to be exported to the cytosol upon dsDNA stimulation, leading to re-expression of otherwise silenced genes (29). We therefore cannot exclude that DDR-signaling in response to ISD is, in part, the consequence of titration of RAD51, or other key factors involved in genome maintenance.

To conclude, our results establish ISDs as DDR inducers. The cGAS-STING pathway primary function is to detect exogenous DNA from pathogens. Some viruses, notably HIV, have evolved to block cGAS activation (44). In this case, the DNA damage pathway may provide a substitute to activate STING and DDR in order to protect infected cells. The cGAS-STING and DNA damage responses show many levels of connections, and we propose that they function in concert as two main gatekeepers in charge of cellular and organism protection. These essential functions in which DNA damage response and innate immunity co-operate in regulating key cellular processes such as proliferation arrest by senescence (14, 16, 30, 31, 37) and inflammaging (45, 46). These connections between DDR and innate immunity were previously described at the molecular level. Both MRE11 (35) and DNA-PK (9, 20) have been proposed to be alternative sensors of cytosolic DNA and activating an inflammatory response. DNA-PK possesses different modes of regulation of innate immunity and anti-viral immunity, both through structural roles and its kinase activity. DNA-PK can either promote (9, 20, 21, 23) or limit (22, 47) cGAS-STING-IFN-I signaling and anti-viral response depending on the type of challenge and pathogens. Future work will provide better understanding of how cells use and coordinate these different modes of DNA sensing in genome maintenance and in innate immunity.

## Experimental procedures

### Cell culture

T98G and THP-1 cells were maintained in culture as previously described (20). HeLa and BJ-h*TERT* cells were maintained in Dulbecco's modified Eagle's medium (DMEM) supplemented with 10% fetal bovine serum (Gibco) and 1% penicillin/streptomycin (Sigma-Aldrich).

### Transfection of ISD

Cells were seeded in 10 mm glass coverslips or 6-well plate 18 h before transfection. The day of transfection, media was carefully removed and 2 μg/ml of ISD was transfected using the Lipofectamine 3000 reagent (Invitrogen) at 1:2 ratios, following the manufacturer’s instructions. 2, 4, 24 or 48 h after transfection, cells were fixed with 2% paraformaldehyde (PFA) or harvested for protein and RNA extraction. Other concentrations of ISD were also tested (see Fig. S1*B*).

### Reagents, drugs and treatments

Hydroxyurea (HU, Merck) was dissolved in water and used at final concentrations of 0.2 mM, 0.5 mM or 2 mM, as indicated in figures. H-151 (from Invivogen, used at 5 μM) was dissolved in DMSO (SIGMA). KU55933 (ATMi) and NU7441 (DNA-PKi) were all from SIGMA, dissolved in DMSO and used at a final concentration of 2 μM.

### Immunofluorescence and microscopy analysis

Cells were seeded on glass coverslips 18 h prior to ISD transfection or HU treatment and fixed with 2% PFA-PBS. PFA fixation was followed by permeabilization in 0,1% Triton X-100 in PBS for 15 to 30 min at room temperature (RT). After blocking in PBS containing 1% BSA (Bovine Serum Albumin) for 1 h at RT, cells were incubated overnight at 4 °C with dilution of primary antibodies in PBS containing 0,1% Tween (PBS-T), 1% BSA. Primary antibody used in immunofluorescence is an anti-phospho-Histone H2A.X (Ser139) (#9718, Cell Signaling, 1:400). Secondary antibody (Alexa Fluor 568 goat anti-Rabbit IgG, #A11036 Thermo Fisher Scientific) incubation was performed for 1 hour at RT. Nuclei were stained with DAPI and coverslips mounted in Vectashield mounting media. Images were acquired at 20X or 40X magnification with an Axio Imager Z2 microscope (Zeiss) with ZEN software (https://www.zeiss.com/microscopy/fr/produits/logiciel/zeiss-zen.html) (blue edition, Zeiss). Images were then processed with Fiji (https://imagej.net/software/fiji/downloads). The intensity of γH2AX fluorescence was measured using CellProfiler software (https://cellprofiler.org/releases).

### Western blots and antibodies

Protein extraction and Western blotting are described in supplemental information. Primary antibodies include anti-ISG15 (ab133346, Abcam), anti-pATM Ser1981 (ab81292, Abcam), anti-ATM (ab201022, Abcam), anti-pTBK1 (5483, Cell Signaling technology), anti-TBK1 (3504, Cell Signaling technology), anti-pSTAT1 (9167, Cell Signaling technology), anti-STAT1 (9172, Cell Signaling technology), anti-pDNA-PK S2056 (ab124918, Abcam), anti-DNA-PK (ab32566, Abcam), anti-TUBULIN (ab6161, Abcam, 1:3000), anti-pSTING (50,907, Cell Signaling Technology) and anti-γH2AX (9718, Cell Signaling Technology), anti-HSP90 (4877, Cell Signaling Technology).

### RNA extraction and gene expression analysis

Methodology and primers are in supporting information.

## Data availability

The data of this article are all available in the article, in its supporting information, and will be shared upon request to the corresponding author.

## Supporting information

This article contains supporting information (8, 20).

## Conflict of interest

The authors declare that they have no conflicts of interest with the contents of this article.
