# Supplementary material for: Interferon stimulatory DNA activates the DNA damage signaling through ATM and DNA-PK sensing
Source: J Biol Chem. 2026 Mar 9;302(5):111362. doi: 10.1016/j.jbc.2026.111362 (PMC13068820; doi:10.1016/j.jbc.2026.111362)
Supplement: Supporting information [file mmc1.pdf]

Supplemental Information

Interferon Stimulatory DNA activates the DNA damage signaling through ATM and DNA-PK sensing

Samira Kemiha [1], Lorena Rejón-Franco [1], Estelle Ghibaudo [1], Roger J. Eloiflin [2], Morgane Chemarin [2], Karim Hawillo [1], Nadine Laguette [2] and Hervé Técher [1,#]

1. Université Côte d'Azur, IRCAN, CNRS, INSERM, Nice, France.

2. IGMM, Université de Montpellier, CNRS, Montpellier, France.

# Correspondence should be addressed to: herve.techer@univ-cotedazur.fr

Contains five additional figures, supplemental experimental procedures and supplemental table 1.

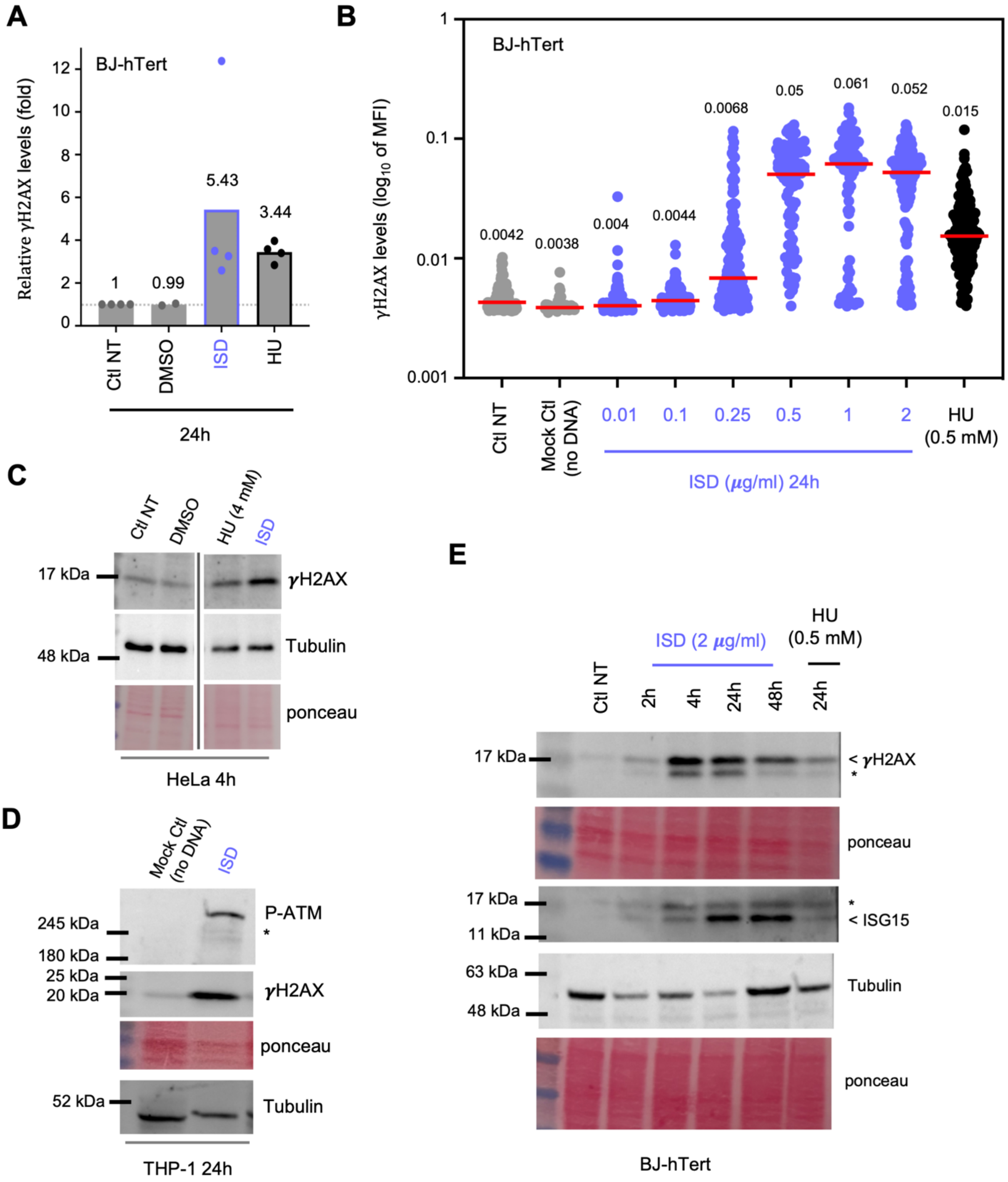

Figure S1. Threshold and timing of activation of DDR upon ISD transfection.
A. BJ-h*TERT* cells were transfected for 24h with 2 μg/ml of ISD and levels of γH2AX were quantified by fluorescent microscopy. Bars are the relative mean intensity from independent experiments (fold change). Each dot is relative intensity obtained from an independent experiment. B. BJ-h*TERT* cells were transfected for 24h with increasing concentrations of ISD. Quantification of nuclear levels of γH2AX are shown as in Fig. 1C. C. HeLa cells were transfected for 4h with 2 μg/ml of ISD and levels of γH2AX were assessed by western blot. Tubulin and ponceau are shown as loading controls. The vertical grey line shows that samples were on different part of the membrane. D. THP-1 cells were transfected for 24h with 2 μg/ml of ISD and levels of γH2AX and phosphorylated ATM (P-ATM) were assessed by western blot. Tubulin and ponceau are shown as loading controls. E. . BJ-h*TERT* cells were transfected in between 2 to 48 hours with ISD (2 μg/ml). Then γH2AX and ISG15 were assessed by western blot. Tubulin and ponceau are loading controls.

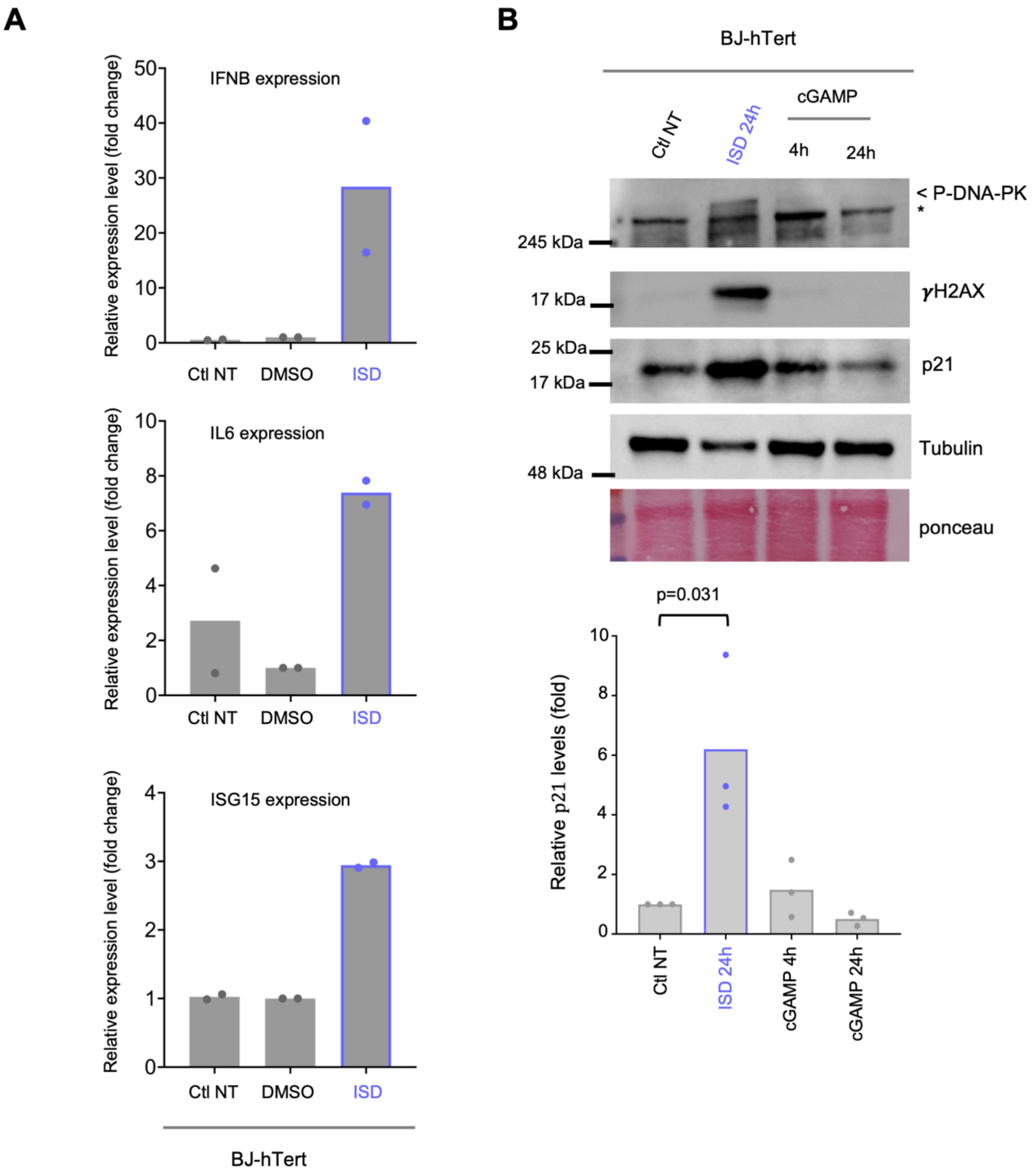

Figure S2. ISD induces the expression of type I IFN-stimulated genes and p21/CDKN1A in BJ-hTERT fibroblasts.

A. BJ-hTERT cells were transfected or not with 2 µg/ml ISD for 24h. IFNB, ISG15, IL6 mRNA levels were quantified by RT–qPCR. Results are the mean of two independent experiments.

B. Upper panel. Representative confirmation of results shown in Fig2A. Bottom panel. Quantification of p21 protein levels from three independent experiments.

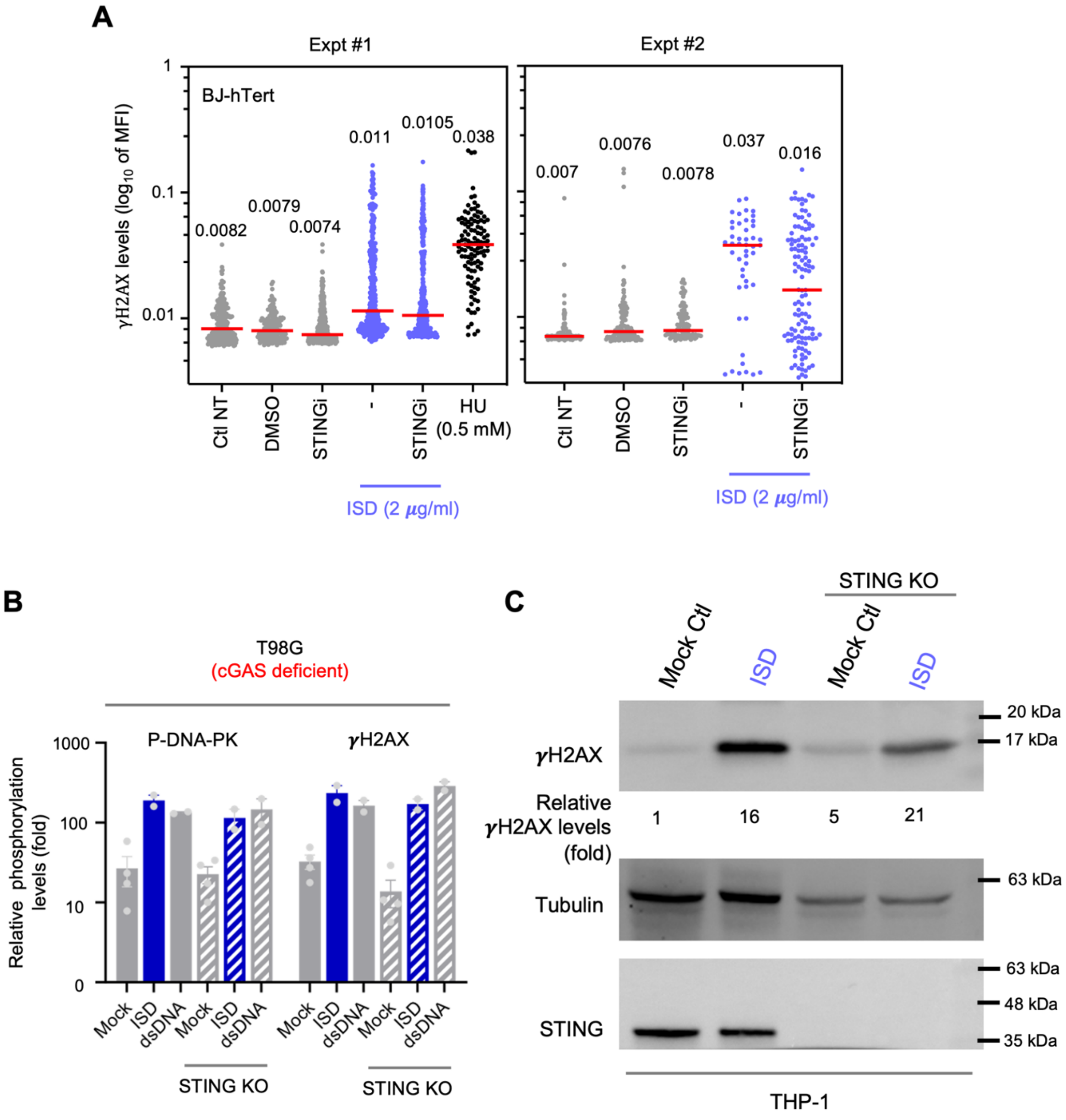

Figure S3. Neither STING inhibition nor STING ablation impact on DDR-signaling induced by ISD.

A. BJ-hTERT cells were transfected with 2 μg/ml of ISD for 24h. Quantification of nuclear levels of γH2AX are shown from a representative experiment in BJ-hTERT cells. We used an overnight treatment (around 16 hours) with 5 μM of STING inhibitor H-151. Results are analyzed and presented as in Fig 1C. Two representative experiments are shown (Expt #1 and #2).

B. Quantifications of wester-blot of main Fig 2B. Results are the mean of two independent experiments.

C. Western blot analysis of γH2AX in THP-1 and THP-1 KO cells. Tubulin is shown as a loading control. Quantification of the of γH2AX signals are shown (normalized to tubulin).

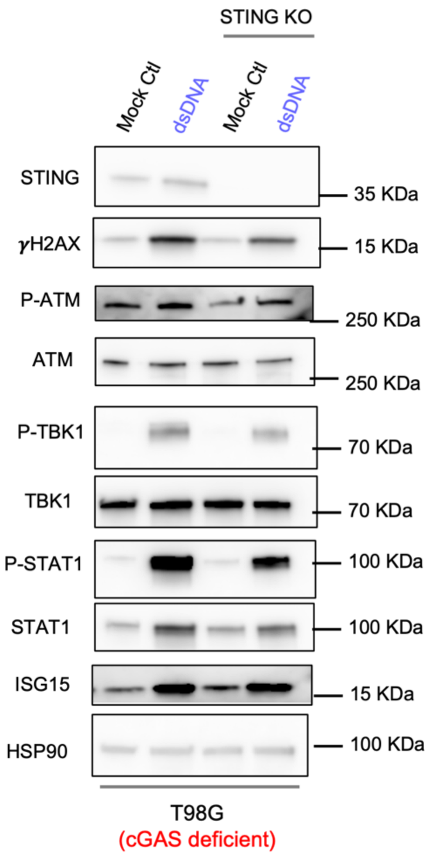

Figure S4. dsDNA activates DDR-signaling independently of the cGAS-STING pathway in T98G cells.

Western blot analysis in WT and STING-/- T98G cells transfected or not with 2 µg/ml of dsDNA (described in references 8 and 20). Results are presented as in Fig 2B.

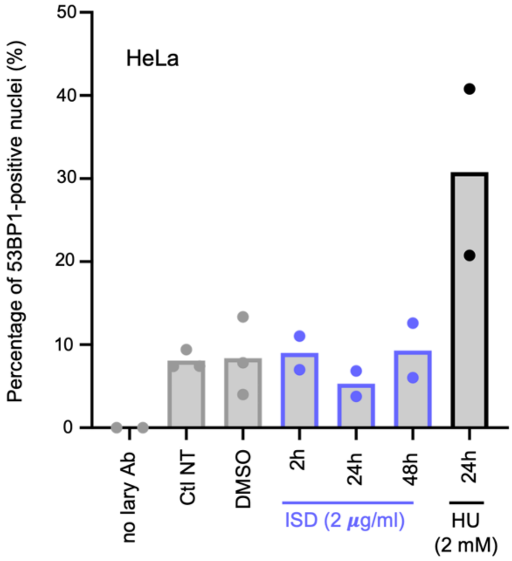

Figure S5. ISD transfection does not lead to 53BP1 foci formation in HeLa cells.
The percentage of 53BP1 positive-cells was assessed by immunofluorescence. Positive cells are cells with 2 or more foci. Bars are the mean of independent experiments. Each dot represent the result of an independent experiment.

Supplemental experimental procedures

Western-blots

HeLa and BJ-h*TERT* cells were seeded in a 6-well plate 18 h before transfection or HU treatment and cells were lyzed in Laemmli buffer (Bio-rad) supplemented with 2,5% β-mercaptoethanol. Samples were incubated with 0,375 units/µL of benzonase (Merck) for 30 min at 37°C. T98G cells were seeded in a 6-well plate 18 h before transfection and were harvested on ice cold phosphate-buffered saline (PBS) using a cell scraper and lysed in 5 packed cell volume (PCV) of TENTG-150 [20 mM tris-HCl (pH 7.4), 0.5 mM EDTA, 150 mM NaCl, 10 mM KCl, 0.5% Triton X-100, 1.5 mM MgCl2, and 10% glycerol] supplemented with 10 mM β-Mercaptoethanol, 0.5 mM phenylmethylsulfonyl fluoride (PMSF) and phosphatase inhibitor (Phosphatase Inhibitor Cocktail 3, Sigma-Aldrich) for 30 min at 4°C. Cell lysates were centrifuged at 12,000g for 30 min at 4°C and supernatants collected. Protein concentration was determined using the Bradford assay (Sigma-Aldrich). Protein samples were prepared in Laemmli buffer, heated at 95°C for 5 min and run on 4-20% Mini-PROTEAN® TGX™ Precast Protein Gels (Bio-Rad) followed by transfer onto nitrocellulose membranes using the Trans-Blot Turbo Transfer System (Biorad). Membranes were incubated with primary antibodies (1:1000 dilution except when indicated) for 1 h at RT or over-night at 4°C. Membranes were incubated with secondary antibodies (Santa Cruz Biotechnology) at 1:5000 dilution for 1 h at RT. Signal was visualized with SuperSignal West Pico PLUS (Thermo Fisher Scientific) or SuperSignal West Femto Maximum Sensitivity Substrate (Thermo Fisher Scientific), and images were acquired on a Fusion Solo camera or Amersham bioluminescence detection imager.

RNA extraction and gene expression analysis

BJ-h*TERT* cells were lysed directly in 6-well plates using 1 mL of TRIzol™ Reagent (Thermo Fisher Scientific), following the manufacturer's instructions (TRIzol Reagent Pub No. MAN0001271). RNA isolation was performed according to the same protocol. Reverse transcription was carried out using the QuantiTect® Reverse Transcription Kit (Qiagen), according to the manufacturer's instructions. For RT-qPCR, a master mix was prepared for each reaction using SYBR® Green Master Mix (Sigma-Aldrich), the primers (synthesized by Sigma-Aldrich), and RNase-free water (Thermo Fisher Scientific). From T98G cells total RNA was extracted with TRIzol reagent (Invitrogen) and analyzed using the protocol described in (8). Relative quantities of the transcripts were calculated using the ΔΔCt method, using either GAPDH, HRPT or β-actin, for normalization in T98G and BJ-hTERT cells, respectively. Primers used are listed below in Supplemental Table 1.

Supplemental Table 1. List of primers used for RT-qPCR.

For BJ-hTERT cell

Gene Name	Forward Primer 5´→ 3´	Reverse Primer 5´→ 3´
β-actin	TGTCCACCTTCCAGCAGATGT	CACCTTCACCGTTCCAGTTTT
ISG15	CGCAGATCACCCAGAAGATCG	TTCGTCGCATTTGTCCACCA
HPRT	GACCAGTCAACAGGGGACAT	AACACTTCGTGGGGTCCTTTTCAC
IL6	GTCAGGGGTGGTTATTGCAT	AGTGAGGAACAAGCCAGAGC
P21	TCACTGTCTTGTACCCTTGTGC	GGCGTTTGGAGTGGTAGAAA
INFβ	AGGACAGGATGAACTTTGAC	TGATAGACATTAGCCAGGAG

For T98G cells

Gene Name	Forward Primer 5´→ 3´	Reverse Primer 5´→ 3´
INFβ	GAATGGGAGGCTTGAATACTGCCT	TAGCAAAGATGTTCTGGAGCATCTC
IL6	GACCCAACCACAAATGCCAG	GTGCCCATGCTACATTTGCC
IL8	CACCGGAAGGAACCATCTCA	TGGCAAAACTGCACCTTCACA
P21	TGAGCCGCGACTGTGATG	GTCTCGGTGACAAAGTCGAAGTT
